# Increased Serum VEGF-B Level Is Associated With Renal Function Impairment in Patients With Type 2 Diabetes

**DOI:** 10.3389/fendo.2022.862545

**Published:** 2022-03-24

**Authors:** Yaping Wei, Shiyu Han, Ruonan Zhou, Pingyuan Xu, Lingyan Zhou, Ziwei Zhu, Yue Kan, Xiaoying Yang, Yingying Xiang, Yue Cao, Yu Jin, Jing Yan, Xizhong Yu, Xin Wang, Wenbin Shang

**Affiliations:** ^1^ Department of Endocrinology, Changzhou Traditional Chinese Medicine Hospital Affiliated to Nanjing University of Chinese Medicine, Changzhou, China; ^2^ Department of Endocrinology, Jiangsu Province Hospital of Chinese Medicine, The Affiliated Hospital of Nanjing University of Chinese Medicine, Nanjing, China; ^3^ Key Laboratory for Metabolic Diseases in Chinese Medicine, First College of Clinical Medicine, Nanjing University of Chinese Medicine, Nanjing, China

**Keywords:** vascular endothelial growth factor B, type 2 diabetes mellitus, diabetic kidney disease, glomerular filtration rate, cystatin C

## Abstract

**Aims/Introduction:**

Renal function impairment related to type 2 diabetes (T2DM) presents serious threat to public health. Previous studies suggest that vascular endothelial growth factor-B (VEGF-B) might contribute to renal injury. Therefore, this study investigated the association of serum VEGF-B level with the risk of renal function impairment in T2DM patients.

**Materials and Methods:**

Serum VEGF-B levels were measured in 213 patients with type 2 diabetes and 31 healthy participants. Participants with type 2 diabetes were further divided into a group of 112 participants with eGFR<90 mL/min/1.73m^2^ and 101 participants with eGFR≥ 90 mL/min/1.73m^2^. Clinical data were collected, and a binary logistic regression model was employed to test the association between potential predictors and eGFR.

**Results:**

Serum VEGF-B levels evaluated in type 2 diabetes patients compared with healthy controls. In patients with type 2 diabetes, serum VEGF-B level was positively correlated with triglyceride, serum creatinine and cystatin C while negatively correlated with HDL-C and eGFR. Binary logistic regression showed that serum VEGF-B level was an independent risk factor of eGFR<90 mL/min/1.73m^2^.

**Conclusions:**

Serum VEGF-B level is associated with renal function impairment in patients with type 2 diabetes and may be a potential drug target for diabetic kidney disease.

## Introduction

With the global prevalence of type 2 diabetes mellitus (T2DM), diabetic kidney disease (DKD) has become the leading cause of end stage renal disease (ESRD), and poses a great burden to diabetes patients and the health care system (1). Progressing renal function impairment characterized by declining glomerular filtration rate (GFR) runs through the course of T2DM, and eventually results in DKD. Multifactorial pathological processes have been proven to be contributors of diabetes-related renal function impairment, including advanced glycation end products (AGEs), ectopic lipid deposition, hemodynamic perturbations and inflammation (2, 3). However, further study is still required to explore the exact mechanisms underlying renal function impairment in T2DM patients.

Vascular endothelial growth factor-B (VEGF-B) is a member of the vascular endothelial growth factor (VEGF) family expressing in multiple organs, such as heart, adipose, muscle, brain, and kidney (4). Different with other members from VEGF family, VEGF-B shows faint angiogenic effect *in vivo*, which triggers a widespread speculation of VEGF-B playing its angiogenic role mainly through its recruitment of other VEGFs (5, 6). However, VEGF-B was reported to regulate fatty acid transport proteins (FATPs) in endothelial cells *via* membrane receptors neuropilin-1(NRP-1) or vascular endothelial growth factor receptor-1 (VEGFR-1), and an improvement of insulin sensitivity along with blood lipid profiles was observed in VEGF-B deficient mice fed with high-fat diet (4, 7). Moreover, the activation of VEGFR-1, one of the VEGF-B receptors, was demonstrated to promote the generation of pro-inflammatory and pro-angiogenic cytokines in macrophages and accelerate the process of inflammatory diseases, for instance, rheumatoid arthritis and retinal injury (8, 9). Seeing the coincidences between VEGF-B biological functions and diabetes-related renal function impairment, several studies reported that VEGF-B could directly impair podocyte insulin sensitivity by promoting ectopic lipid accumulation in podocytes and cause the occurrence of DKD in various diabetic mouse models (10, 11). Furthermore, a newly produced anti-VEGFB/IL22 fusion protein was found to be capable to ameliorate renal dysfunction in db/db mice recently (11).

Although evidences from animal experiments suggest the vital role of VEGF-B in DKD occurrence, the relationship between circulating VEGF-B level and renal function impairment in T2DM patients still remains unclear. In this study, we tested the serum VEGF-B levels of 213 T2DM patients as well as 31 healthy participants, and analyzed the potential link between serum VEGF-B levels and renal function of patients with T2DM. Our results would provide a better insight into the role of VEGF-B in the pathogenesis of diabetic renal impairment as well as the intervention of diabetic kidney disease.

## Methods

### Subjects

31 healthy participants were recruited from the health examination center of the affiliated hospital of Nanjing university of Chinese medicine. None of the healthy participants had known metabolic disorders, kidney injury or other kind of diseases. 213 T2DM patients were recruited from the department of endocrinology of the affiliated hospital of Nanjing university of Chinese medicine. All participants with T2DM satisfied the world health organization 1999 criteria when diagnosed (12). This study was approved by the medical research ethics committee of the affiliated hospital of Nanjing university of Chinese medicine and carried out under the principles of the Declaration of Helsinki. Written informed consents were obtained from all participants.

### Clinical Data Collection

All participants were admitted to the department of endocrinology. Detailed medical history and physical examination data, including gender, age, medicine usage, resting blood pressure, weight and height were obtained upon admission. Venous blood and midstream urine samples were collected at 6:00 a.m. from participants after 12 hours of fasting. The examination of total cholesterol (TC), triglyceride (TG), low density lipoprotein (LDL-C), high density lipoprotein (HDL-C), fasting blood-glucose (FBG), fasting C peptide (FCP), glycosylated hemoglobin (HbA1c), serum creatinine (SCr), blood urea nitrogen (BUN), Cystatin C (CysC), urine albumin-to-creatinine ratio (UACR) and 24-hour urine total protein (UTP) were performed at laboratory center of the affiliated hospital of Nanjing university of Chinese medicine. Body mass index (BMI) = body weight (kg)/the square of body height (m^2^). Homeostasis model of assessment for insulin resistance (HOMA-IR) = fasting glucose (mmol/L)╳fasting insulin (mU/L)/22.5. Estimated glomerular filtration rate (eGFR) was calculated according to CKD-EPI2012scr-cys.

### Serum VEGF-B Measurement

Fresh blood samples were stood at room temperature (22°C) for 30min and then centrifuged at 3000rpm for 10 min. The supernatant were collected as serum samples and stored at -80°C. Serum VEGF-B values were determined by human enzyme-linked immunosorbent assay (ELISA) kits (CB5521, Biorbyt, UK).

### Statistical Analysis

Statistical analyses were performed using SPSS 25.0. All data were represented as mean ± standard deviation (SD), median with interquartile range (IQR) or percentage, as appropriate. Data that were not normally distributed, including Diabetes duration, FBG, HbA1c, HOMA-IR, Triglyceride, Total cholesterol, HDL-C, LDL-C, Serum creatinine, Blood urea nitrogen, Cystatin C, UACR, UTP, eGFR, Serum VEGF-B were logarithmically transformed before analysis. ANOVA and Mann-Whitney U tests were used for continuous variables distributed normally and asymmetrically, respectively. Chisquared test (*χ* ^2^) was employed for comparisons of categorical variables. The relationships between clinical indicators were examined using *Pearson* correlation, *Spearman* correlation or partial correlation. Binary logistic regression analysis was used to test the association between potential predictors and eGFR. A p-value of <0.05 was indicated statistically significant.

## Results

### Serum VEGF-B Level Elevates in T2DM Patients

**Figure 1:**
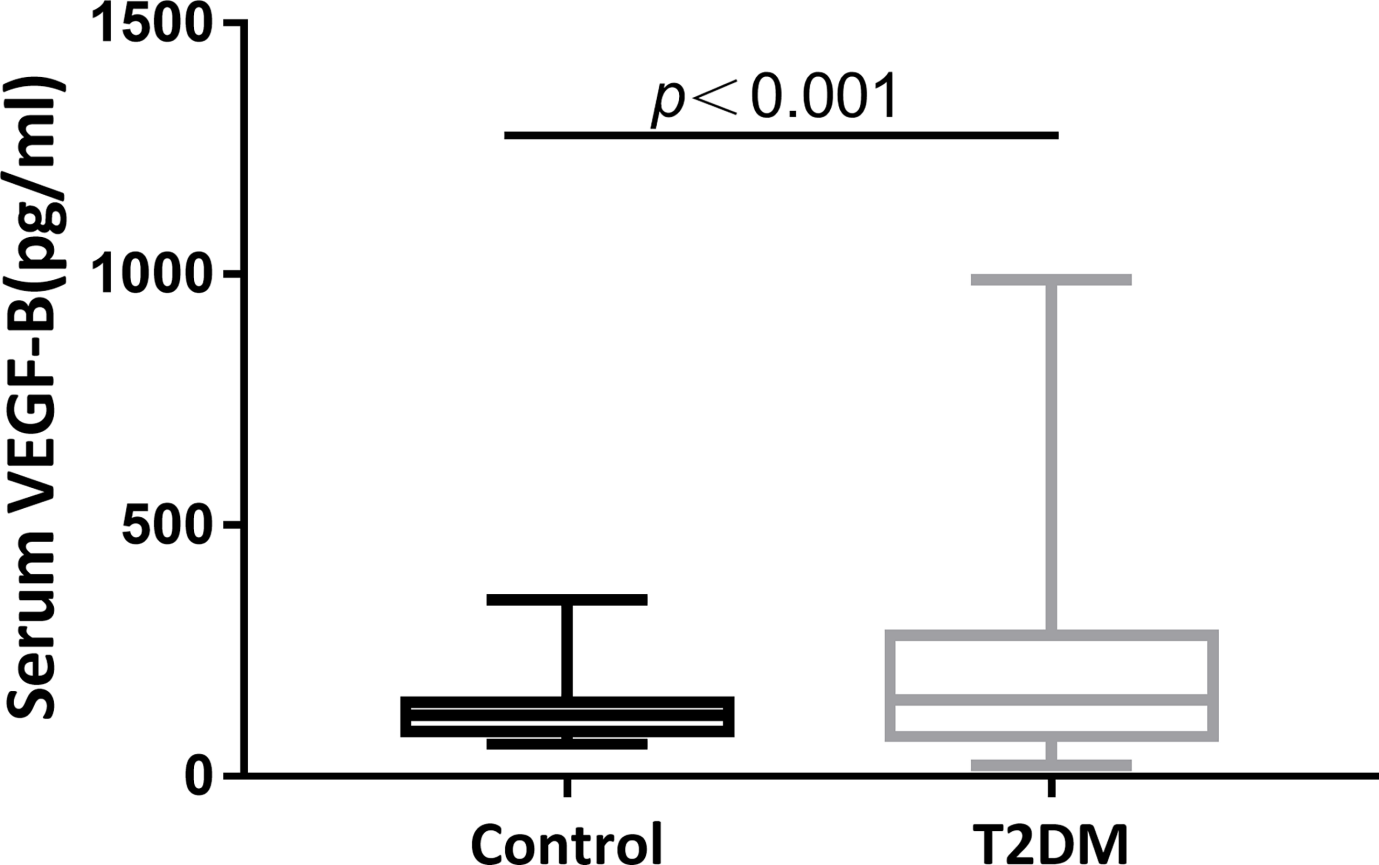
Serum VEGF-B level elevates in T2DM patients. A significantly increase of serum VEGF-B levels in T2DM patients compared to healthy control subjects was observed (*p *< 0.001).

The study recruited 244 eligible participants, including 213 patients explicitly diagnosed with T2DM at baseline and 31 healthy subjects seeking for routine examination. Fasting blood samples were collected from all participants and serum VEGF-B levels were tested. As shown in **Figure 1**, compared to the healthy controls, a significantly elevated level of serum VEGF-B was observed in T2DM patients (*p*<0.001).

### Baseline Characteristics of Participants With T2DM

Based on the accepted eGFR cutoff value (90 mL/min/1.73m^2^), we further divided the enrolled T2DM patients into a group of 112 participants with eGFR<90 mL/min/1.73m^2^ and 101 participants with eGFR≥ 90 mL/min/1.73m^2^ (13). Gender distribution, drinking history, smoking history, BMI, SBP, HbA1c, FPG, HOMA-IR, TC, TG, HDL-C, LDL-C, UCR and UTP were similar between the groups (all *p*>0.05). However, the eGFR<90 mL/min/1.73m^2^ group had older age (*p*<0.001), longer diabetes duration (*p*=0.004), lower DBP (*p*=0.003), as well as worse renal dysfunction characterized by higher levels of SCr (*p*<0.001), BUN (*p*<0.001), CysC (*p*<0.001) and UACR (*p*=0.005). Most importantly, eGFR<90 mL/min/1.73m^2^ group also showed a significantly increased level of serum VEGF-B (*p*=0.002) (**Table 1**). Upon the usage of medications, no significant difference was observed between 2 groups on the usage of metformin, sulfonylureas, pioglitazone, glucosidase inhibitors, GLP1rA (glucagon-like peptide 1 receptor agonists), DPP4 inhibitors, SGLT2 inhibitors, statins, insulin and ACEI/ARB (all *p*>0.05). But, a higher percentage of patients using beta-blockers (*p*=0.007) and Calcium channel blockers (*p*=0.039) in eGFR<90 mL/min/1.73m^2^ group was showed by Chisquared test (**Table 2**).

**Table 1:** Anthropometric characteristics, clinical characteristics and VEGF-B levels.

Characteristic	eGFR<90 mL/min/1.73m^2^(n = 112)	eGFR≥ 90 mL/min/1.73m^2^(n = 101)	*p-*value
Female, n (%)	39 (34.82)	36 (35.64)	0.090^c^
Age (years)	66.00 (57.25, 72.00)	53.00 (46.00, 58.50)	<0.001^b*^
Smoking, n (%)	25 (22.32)	22 (21.78)	0.925^c^
Drinking, n (%)	12 (10.71)	20 (19.80)	0.064^c^
^△^Diabetes duration (years)	0.99 ± 2.06	1.77 ± 1.86	0.004^a*^
BMI (kg/m^2^)	25.16 ± 3.23	25.42 ± 3.71	0.590^a^
^△^Systolic pressure (mmHg)	4.88 ± 0.13	4.91 ± 0.14	0.236^a^
Diastolic pressure (mmHg)	75.46 ± 10.35	79.96 ± 11.60	0.003^a*^
^△^FBG (mmol/L)	1.93 ± 0.35	1.91 ± 0.37	0.692^a^
^△^HbA1c (%)	2.17 ± 0.25	2.16 ± 0.26	0.670^a^
^△^HOMA-IR	1.11 ± 0.80	1.20 ± 0.86	0.424^a^
^△^Triglyceride (mmol/L)	0.55 ± 0.64	0.57 ± 0.61	0.281^a^
^△^Total cholesterol (mmol/L)	1.50 ± 0.24	1.47 ± 0.26	0.916^a^
^△^HDL-C (mmol/L)	0.16 ± 0.24	0.13 ± 0.27	0.262^a^
^△^LDL-C (mmol/L)	1.02 ± 0.32	1.00 ± 0.37	0.544^a^
^△^Serum creatinine (umol/L)	4.03 ± 0.21	4.33 ± 0.33	<0.001^a*^
^△^Blood urea nitrogen (mmol/L)	1.68 ± 0.27	1.88 ± 0.33	<0.001^a*^
^△^Cystatin C (mg/L)	-0.21 ± 0.13	0.20 ± 0.25	<0.001^a*^
^△^UACR (mg/g)	2.36 ± 1.22	2.94 ± 1.88	0.005^a*^
^△^UTP (mg/24h)	4.21 ± 0.98	4.43 ± 1.44	0.465^a^
^△^eGFR (mL/min/1.73m^2^)	4.66 ± 0.10	4.22 ± 0.34	<0.001^a*^
^△^Serum VEGF-B (pg/mL)	4.80 ± 0.77	5.16 ± 0.84	0.002^a*^

BMI, body mass index; FBG, fasting blood-glucose; HDL-C, high density lipoprotein; LDL-C, low density lipoprotein; UACR, urine albumin-to-creatinine ratio; UTP, 24-hour urine total protein; eGFR, estimated glomerular filtration rate.

^△^Log-transformed before analysis.

*Significance, p < 0.05.

^a^Student’s t-test.

^b^Mann-Whitney U test.

^c^Chisquared test.

**Table 2:** Medicine usage of T2DM patients recruited.

Use of medications	eGFR<90 mL/min/1.73m^2^	eGFR≥ 90 mL/min/1.73m^2^	*p-*value
	(n = 112)	(n = 101)	
Metformin, %	46.08%	44.55%	0.296
Sulfonylureas, %	27.45%	27.72%	0.167
Pioglitazone, %	5.88%	5.94%	0.551
Glucosidase inhibitors, %	24.51%	24.75%	0.440
GLP1rA, %	2.94%	2.97%	0.898
DPP4 inhibitors, %	25.49%	24.75%	0.115
SGLT2i, %	11.76%	11.88%	0.741
Statins, %	23.53%	23.76%	0.077
Insulin, %	65.69%	65.35%	0.911
ACEI/ARB, %	27.45%	27.72%	0.129
Beta-blockers, %	36.61%	19.80%	0.007^*^
CCB, %	35.71%	22.77%	0.039^*^
Diuretics, %	3.92%	3.96%	0.095

GLP1rA, glucagon-like peptide 1 receptor agonists; SGLT2i, sodium-glucose co-transporter 2 inhibitors; CCB, Calcium Channel Blockers.

^*^Significance, p < 0.05.

### Serum VEGF-B Level Significantly Associates With Renal Function Indicators in T2DM Patients

**Figure 2:**
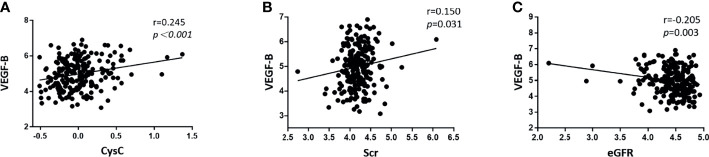
Scatter diagrams showing the significant partial correlations between serum VEGF-B levels and renal function indicators in T2DM patients after adjusting for gender, age, smoking history, drinking history, diabetes duration and BMI. **(A)** The serum VEGF-B level was positively correlated with Cystatin C (r=0.245, *p* < 0.001); **(B)** The serum VEGF-B level was positively correlated with Scr (r=0.150, *p*=0.031); **(C)** The serum VEGF-B level was negatively correlated with eGFR (r=-0.205, *p*=0.003). All data was log-transformed before analysis.

As shown in **Table 3**, in T2DM patients, serum VEGF-B level was positively correlated with triglyceride (r=0.172, *p*=0.013), serum creatinine (r=0.150, *p*=0.031) and cystatin C (r=0.245, *p*<0.001) while inversely correlated with HDL-C (r=-0.138, *p*=0.047) and eGFR (r=-0.205, *p*=0.003) after adjusting gender, age, smoking history, drinking history, diabetes duration and BMI. However, although partial correlation confirmed that VEGF-B level significantly associates with several renal function indicators (**Figures 2A–C**), no significant correlation was observed between serum VEGF-B and UACR nor UTP (both *p*>0.05).

**Table 3:** The correlation of serum VEGF-B (log-transformed) with clinical indicators in T2DM patients.

	Serum VEGF-B level	
	r	*p-*value
Gender	0.013	0.853^a^
Age	0.070	0.308^b^
Smoking	-0.080	0.248^a^
Drinking	-0.003	0.962^a^
^△^Diabetes duration	0.130	0.059^a^
BMI	0.000	0.994^a^
^△^Systolic pressure	0.019	0.790^c^
Diastolic pressure	0.056	0.422^c^
^△^FBG	0.098	0.160^c^
^△^HbA1c	0.056	0.420^c^
^△^HOMA-IR	0.050	0.472^c^
^△^Triglyceride	0.172	0.013^c*^
^△^Total cholesterol	0.062	0.371^c^
^△^HDL-C	-0.138	0.047^c*^
^△^LDL-C	0.121	0.083^c^
^△^Serum creatinine	0.150	0.031^c*^
^△^Blood urea nitrogen	0.026	0.713^c^
^△^Cystatin C	0.245	<0.001^c*^
^△^UACR	0.025	0.721^c^
^△^UTP	-0.030	0.668^c^
^△^eGFR	-0.205	0.003^c*^

BMI, body mass index; FBG, fasting blood-glucose; HDL-C, high density lipoprotein; LDL-C, low density lipoprotein; UACR, urine albumin-to-creatinine ratio; UTP, 24-hour urine total protein; eGFR, estimated glomerular filtration rate.

^△^Log-transformed before analysis.

^*^Significance, p < 0.05.

a Pearson correlation.

b Spearman correlation.

c Partial correlation analysis adjusted for gender, age, smoking history, drinking history, diabetes duration and BMI.

### Regression Model of eGFR in T2DM Patients

**Figure 3:**
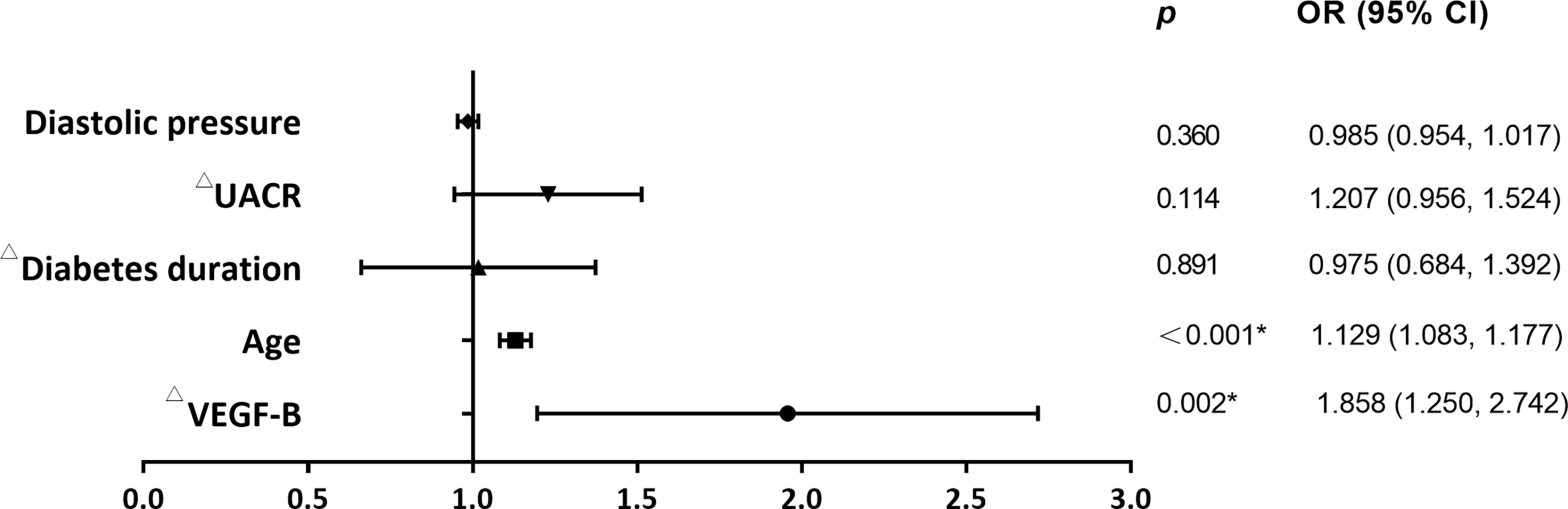
Serum VEGF-B level is an independent risk factor for eGFR<90 mL/min/1.73m^2^. As shown in the forest plots, binary logistic regression demonstrated that serum VEGF-B level and age are independent risk factors for eGFR<90 mL/min/1.73m^2^. ^*^Significance, *p*<0.05. ^△^Log-transformed before analysis.

We further employed binary logistic regression analysis to analyze the associations between serum VEGF-B and the risk of eGFR<90 mL/min/1.73m^2^. We found that serum VEGF-B level (*p*=0.002) was significant in predicting eGFR<90 mL/min/1.73m^2^, even after controlling age, UACR, diabetes duration and diastolic pressure (based on the results from **Table 1**). Of the risk factors above, age (*p*<0.001) showed a significant, positive relationship with eGFR<90 mL/min/1.73m^2^ as well (**Table 4** and **Figure 3**).

**Table 4:** Risk factors of eGFR<90 mL/min/1.73m^2^ (Binary logistic regression).

Variables	B	SE	Wald	*p*-value	OR	95% CI	
^△^VEGF-B	0.619	0.202	9.376	0.002^*^	1.858	1.250	2.762
Age	0.121	0.021	32.468	<0.001^*^	1.129	1.083	1.177
Diabetes duration	-0.025	0.181	0.019	0.891	0.975	0.684	1.392
^△^UACR	0.188	0.119	2.499	0.114	1.207	0.956	1.524
^△^Diastolic pressure	-0.015	0.016	0.837	0.360	0.985	0.954	1.017

Reference category: IC negative.

OR, odds ratio; CI, confidence interval for the odds ratio.

^△^Log-transformed before analysis.

## Discussion

Diabetic kidney disease is one of the leading complications of type 2 diabetes. Currently, the mechanisms underlying T2DM-related renal function impairment remains unclear, and the strategies of DKD prediction are still limited (14). In this study, we assessed whether serum VEGF-B, a biomarker from vascular endothelial growth factor family, was associated with progression of diabetes-related renal function impairment in 213 patients with established T2DM. Our results showed that elevated level of VEGF-B in T2DM patients was significantly correlated with markers of lipid metabolism and glomerular function. Regression model also indicated that serum VEGF-B was one of the independent risk factors of eGFR<90 mL/min/1.73m^2^ in T2DM patients.

Previously, several studies have observed increased VEGF-B levels in DKD patients compared to healthy controls (10, 11). Our study further proved that, not only in DKD patients, just in T2DM population (only 28 of 213 T2DM patients fit the diagnostic criteria of DKD), a significantly rising level of serum VEGF-B could be marked. This may provide evidence for the hypothesis that VEGF-B might be a cause for T2DM-related renal function impairment rather than a result. A genome-wide association study demonstrated that the up-regulation of *vegf-b* is related to chronic kidney disease, T2DM, hypertension and hyperlipidemia (15). In animal models of diabetes, VEGF-B increment was found in various type of cells, such as choroidal cells and podocytes (10, 16). Furthermore, *vegf-b* overexpression impaired whereas *vegf-b* knockout rescued the insulin sensitivity and blood lipid profile of gene-edited mouse models (7, 17). Hence, we can assume that VEGF-B may be a future drug target of T2DM and the complications related with huge potential. In addition, a cross-sectional study involving 45 patients with newly diagnosed T2DM also showed an elevation of circulating VEGF-B level in newly diagnosed T2DM patients compared with healthy subjects, and the study exhibited a significantly correlation between circulating VEGF-B and markers of glucose metabolism as well (18). However, in our study, no significant correlation between serum VEGF-B and FBP nor HOMA-IR was observed, which may on account of the anti-diabetic drug usage. We will further expand the sample size and refine the groups to see if more evidence could be provided.

By correlation analysis, we also demonstrated that serum VEGF-B in T2DM patients was correlated with triglyceride and HDL-C, besides renal function markers. Consistent with our findings, Ye etc. found that circulating VEGF-B level was positively correlated with HDL-C and negatively correlated with eGFR in patients with non-alcoholic fatty liver disease (19). Disorder of lipid metabolism is an acknowledged contributor of diabetes-related renal function impairment (20). It has been proven that VEGF-B could destroy glomerular filtration barrier by inducing ectopic lipid deposition in podocyte, and VEGF-B antibody injection prevented renal lipotoxicity and further improved the renal function of db/db mice (10). Our results confirmed that serum VEGF-B is positively correlated with triglyceride and HDL-C while negatively correlated with eGFR, which supports the point that VEGF-B affects renal function of T2DM patients at least partially *via* regulating lipid metabolism.

It is well known that the classic characteristics of DKD are descending GFR and progressing albuminuria. However, epidemiological investigation exhibits a gradually increasing incidence rate of chronic kidney disease with normoalbuminuria in diabetic patients, especially type 2 diabetes, and the disease was recently referred to as normoalbuminuric diabetic kidney disease (NADKD) (21). Our data also showed that after grouping T2DM patients by eGFR, no difference could be seen in UTP between eGFR≥ 90 mL/min/1.73m^2^ group and eGFR<90 mL/min/1.73m^2^ group (the slight decline of UACR in eGFR<90 mL/min/1.73m^2^ group may depend on the increase of creatinine). This could be partially explained by the limited sample size, but also, the phenomenon was consistent with the epidemic of NADKD in T2DM. Moreover, traditionally, albuminuria and diabetic retinopathy are important components of early DKD prediction and diagnosis. But increasing studies point out that substantial numbers of T2DM patients with DKD do not have retinopathy nor abnormal albuminuria, and normoalbuminuria T2DM patients with low GFR (<60 mL/min/1.73m^2^) are more likely to show diffuse lesions, nodular lesions, tubulointerstitial lesions or vascular lesions compared to those with preserved GFR (≥60 mL/min/1.73m^2^) (22, 23). And as such, more sensitive indicators are urgently needed for early diagnosis and intervention of DKD. In our research, we found that serum VEGF-B level in T2DM patients was positively correlated with SCr and CysC, while negatively correlated with eGFR and is an independent predictor of eGFR<90 mL/min/1.73m^2^. Interestingly, no significant correlation between serum VEGF-B and UTP nor UACR was observed, which suggested that the relationship between serum VEGF-B level and renal function impairment in T2DM patients was independent of albuminuria, and the VEGF-B up regulation is likely to be a new mechanism underlying NADKD in T2DM patients.

Overall, in this study, we demonstrated that T2DM patients exhibited an elevated serum VEGF-B level, which was associated with renal function impairment. The increased serum VEGF-B level was an independent risk factor of eGFR<90 mL/min/1.73m^2^ in patients with type 2 diabetes and it was irrelevant to albuminuria. However, certain limitations of this study should be noted. Firstly, owing to the difficulty in obtaining renal samples from T2DM patients, VEGF-B levels in serum were tested only, thus we are unable to trace the accurate source of the increasing VEGF-B. Secondly, the persuasion as well as the reproducibility of our findings might be blunted by the limited sample size. Current chronic kidney disease nomenclature used by KDIGO is based on eGFR (G1-G5) along with albuminuria (A1-A3), but most of our cases were restricted at G1 and G2 stage, which made it far too difficult to further stage the patients by KDIGO standard. 90 mL/min/1.73m^2^ is an eGFR cutoff value widely accepted by guidelines (13, 24). eGFR<90 mL/min/1.73m^2^ usually indicates renal function impairment. By dividing T2DM patients into eGFR≥ 90 mL/min/1.73m^2^ group and eGFR<90 mL/min/1.73m^2^ group, we found a strong link between serum VEGF-B level and renal function impairment in T2DM patients. However, we will further expand the sample size in the future to explore the role of serum VEGF-B level in different stages of DKD. Lastly, as a cross-sectional research, this study was constrained in inferring causal relationships, and usage of medications was unavoidable in patients recruited. Although Chisquared test showed no significant differences in the usage of RAS inhibitors, SGLT2 inhibitors and statins between eGFR≥ 90 mL/min/1.73m^2^ group and eGFR<90 mL/min/1.73m^2^ group, which sharpens our observations, future studies designed in cohorts are still required to confirm the findings and to determine the role of VEGF-B in DKD pathology as well as whether serum VEGF-B level could be used for early prediction of DKD or NADKD.

## Data Availability Statement

The raw data supporting the conclusions of this article will be made available by the authors, without undue reservation.

## Ethics Statement

The studies involving human participants were reviewed and approved by the medical research ethics committee of the affiliated hospital of Nanjing university of Chinese medicine. The patients/participants provided their written informed consent to participate in this study.

## Author Contributions

In this study, YW and WS designed the project, analyzed the data and wrote portions of the manuscript. SH, RZ, PX, LZ, ZZ, YK, XYY, YX, YC, and XW performed the collection of samples and data. YJ, JY and XZY performed the measurement of serum VGEF-B. WS guided the project and wrote portions of the manuscript. All authors are in agreement with the content of the manuscript, and the authors declare no conflict of interest. All authors contributed to the article and approved the submitted version.

## Funding

The present study was supported by the open projects of the discipline of Chinese Medicine of Nanjing University of Chinese Medicine supported by the subject of academic priority discipline of Jiangsu higher education institutions (ZYX03KF058).

## Conflict of Interest

The authors declare that the research was conducted in the absence of any commercial or financial relationships that could be construed as a potential conflict of interest.

## Publisher’s Note

All claims expressed in this article are solely those of the authors and do not necessarily represent those of their affiliated organizations, or those of the publisher, the editors and the reviewers. Any product that may be evaluated in this article, or claim that may be made by its manufacturer, is not guaranteed or endorsed by the publisher.
